# The Impact of Generative Conversational Artificial Intelligence on the Lesbian, Gay, Bisexual, Transgender, and Queer Community: Scoping Review

**DOI:** 10.2196/52091

**Published:** 2023-12-06

**Authors:** Nicola Luigi Bragazzi, Andrea Crapanzano, Manlio Converti, Riccardo Zerbetto, Rola Khamisy-Farah

**Affiliations:** 1 Laboratory for Industrial and Applied Mathematics Department of Mathematics and Statistics York University Toronto, ON Canada; 2 Department of Counseling San Francisco State University San Francisco, CA United States; 3 Department of Mental Health Local Health Unit ASL Napoli 2 Nord Naples Italy; 4 GESTALT Study Center Milano Italy; 5 Azrieli Faculty of Medicine Bar-Ilan University Safed Israel

**Keywords:** generative conversational artificial intelligence, chatbot, lesbian, gay, bisexual, transgender, and queer community, LGBTQ, scoping review, mobile phone

## Abstract

**Background:**

Despite recent significant strides toward acceptance, inclusion, and equality, members of the lesbian, gay, bisexual, transgender, and queer (LGBTQ) community still face alarming mental health disparities, being almost 3 times more likely to experience depression, anxiety, and suicidal thoughts than their heterosexual counterparts. These unique psychological challenges are due to discrimination, stigmatization, and identity-related struggles and can potentially benefit from generative conversational artificial intelligence (AI). As the latest advancement in AI, conversational agents and chatbots can imitate human conversation and support mental health, fostering diversity and inclusivity, combating stigma, and countering discrimination. In contrast, if not properly designed, they can perpetuate exclusion and inequities.

**Objective:**

This study aims to examine the impact of generative conversational AI on the LGBTQ community.

**Methods:**

This study was designed as a scoping review. Four electronic scholarly databases (Scopus, Embase, Web of Science, and MEDLINE via PubMed) and gray literature (Google Scholar) were consulted from inception without any language restrictions. Original studies focusing on the LGBTQ community or counselors working with this community exposed to chatbots and AI-enhanced internet-based platforms and exploring the feasibility, acceptance, or effectiveness of AI-enhanced tools were deemed eligible. The findings were reported in accordance with the PRISMA-ScR (Preferred Reporting Items for Systematic Reviews and Meta-Analyses extension for Scoping Reviews).

**Results:**

Seven applications (HIVST-Chatbot, TelePrEP Navigator, Amanda Selfie, Crisis Contact Simulator, REALbot, Tough Talks, and Queer AI) were included and reviewed. The chatbots and internet-based assistants identified served various purposes: (1) to identify LGBTQ individuals at risk of suicide or contracting HIV or other sexually transmitted infections, (2) to provide resources to LGBTQ youth from underserved areas, (3) facilitate HIV status disclosure to sex partners, and (4) develop training role-play personas encompassing the diverse experiences and intersecting identities of LGBTQ youth to educate counselors. The use of generative conversational AI for the LGBTQ community is still in its early stages. Initial studies have found that deploying chatbots is feasible and well received, with high ratings for usability and user satisfaction. However, there is room for improvement in terms of the content provided and making conversations more engaging and interactive. Many of these studies used small sample sizes and short-term interventions measuring limited outcomes.

**Conclusions:**

Generative conversational AI holds promise, but further development and formal evaluation are needed, including studies with larger samples, longer interventions, and randomized trials to compare different content, delivery methods, and dissemination platforms. In addition, a focus on engagement with behavioral objectives is essential to advance this field. The findings have broad practical implications, highlighting that AI’s impact spans various aspects of people’s lives. Assessing AI’s impact on diverse communities and adopting diversity-aware and intersectional approaches can help shape AI’s positive impact on society as a whole.

## Introduction

### The Lesbian, Gay, Bisexual, Transgender, and Queer Community

Despite recent significant strides toward acceptance, inclusion, and equality, members of the lesbian, gay, bisexual, transgender, and queer (LGBTQ) community still face alarming mental health disparities, being almost 3 times more likely to experience depression, anxiety, and suicidal thoughts than their heterosexual counterparts [1,2].

This range of unique psychological issues is the result of the challenges associated with navigating and affirming their LGBTQ identities (fear of not being accepted, family and peer rejection, exclusion, isolation, bullying, stigmatization, and societal discrimination) [1,2]. Personal experiences and individual factors, including lack of resilience and inadequate coping skills, can contribute to shaping the response to external stressors and psychological challenges [3] such as internalized homophobia or transphobia—owing to societal norms, prejudices, and negative stereotypes, some LGBTQ individuals internalize feelings of shame or self-hatred about their sexual orientation or gender identity or expression [4]. This can lead to mental health issues, which, besides affective disorders and suicidality, include low self-esteem, body dissatisfaction, eating disorders, substance use, violence, and microaggressions, especially among youth [5]. Multiple or intersecting identities, such as those simultaneously belonging to sexual, gender, and ethnic minorities; being from a low socioeconomic status; or living with disabilities, can lead to further discrimination [6].

Mental health professionals should be culturally competent and understand the unique challenges faced by the LGBTQ community to provide essential support and guidance. Promoting inclusivity, fostering acceptance, and advocating for equal rights can contribute to a more supportive and safer environment for LGBTQ individuals and help mitigate their psychological challenges [7,8].

### Generative Conversational Artificial Intelligence

Generative conversational artificial intelligence (AI) refers to the latest advancements in the field of AI and, more specifically, to a type of AI technology designed to generate humanlike text-based conversations [9] mimicking human conversation patterns [9]. Generative conversational AI systems can react to user input by generating contextually relevant, coherent responses in the form of text that reads as if it had been written by a human, from simple answers to more complex, engaging, and contextually rich interactions. Another characteristic is context awareness—generative conversational AI can consider the context of the ongoing conversation to provide appropriate responses, maintaining a natural flow of dialogue [10].

The rapid development of generative conversational AI has been possible because of several interconnected factors, including technological advancements, the latest findings of scholarly research, availability of increasingly vast amounts of data, collaboration and sharing of AI models and knowledge within the open-source community, and increasing user demand.

Recently, generative conversational AI has become even more prominent owing to the launch of ChatGPT, a large language model (LLM)–based chatbot that has gained remarkable popularity.

### Generative Conversational AI and Health Care

In the health care arena, generative conversational AI can have numerous applications, including chatbots and internet-based assistants, which are computerized, highly automated programs that can select and provide different interventional paths according to participants’ responses, from real-time personalized health promotion and prevention to screening, treatment options, and referral to health care providers for further management. They have several advantages when compared with classic and conventional tools: they can reach people with low access to health care services, including socially vulnerable, marginalized communities, and offer affordable and convenient access. Chatbots and internet-based assistants [11] can enhance the connection between individuals, organizations, and health care providers by handling delicate and sensitive matters without the need for direct human engagement. Furthermore, they help create a sense of protection from preconceptions in users’ minds.

In contrast, users might seek clarification regarding the accuracy and safety of the information presented by chatbots because of their nonhuman nature. Nonetheless, compared with static digital tools such as smartphone apps, chatbots enhance user satisfaction and engagement, which is especially valuable for individuals with a less favorable view of technology, aiding them in accessing necessary information and, consequently, contributing to bridging the digital gap and fostering social inclusiveness. The acceptability of chatbots has been found to correlate with their ability to offer simple conversational interactions centered on key content and mimicking human language [12]. In addition, the chatbot’s capacity to motivate users while allowing them to maintain control over interactions, as well as its effectiveness in delivering assistance and information, play a significant role in its positive reception. Conversely, when emotional support is a central aspect of the conversation, the use of cognitive features is discouraged as it could potentially be perceived as awkward. “Emotional AI” or affective computing through speech or face recognition attempts to bridge this gap.

### Generative Conversational AI, Health Care, and the LGBTQ Community

The LGBTQ community could benefit from the latest advancements in the field of AI in terms of accessible, fair, inclusive support as well as safe spaces for individuals seeking information and advice without fear of judgment. Chatbots and AI-driven platforms could assist LGBTQ individuals who, as previously mentioned, may struggle with disclosing personal information or concerns to others because of privacy concerns and, therefore, might be hesitant to seek help because of stigmatization, discrimination, or lack of understanding from others.

Chatbots offer a level of anonymity, allowing individuals to discuss sensitive topics openly without revealing their identity, which can be particularly important for those not yet out or living in less accepting environments. Furthermore, AI systems are available around the clock, providing continuous access to information, resources, and support, which is highly valuable for individuals who need assistance during nontraditional hours or in times of crisis. In addition, generative conversational AI can be programmed to provide accurate, evidence-based, culturally sensitive, tailored, and relevant information based on users’ unique identities and needs. This ensures that the guidance and resources offered are applicable to the experiences and challenges of the LGBTQ community, thereby promoting well-informed decision-making. Furthermore, in addition to acting as a “resource navigator,” generative conversational AI may reduce isolation, creating a sense of connection and community by offering opportunities for engagement, discussion, and interaction.

However, empowering the LGBTQ community through generative conversational AI, in addition to its significant benefits, could be challenging and even have detrimental effects (Textbox 1). The ethical implications of AI encompass concerns about biases, privacy, and the potential for overreliance on technology, which becomes even more relevant in particular in mental health scenarios or when dealing with socially vulnerable, marginalized groups. It is imperative to address bias in AI, protect privacy in data use, and strike a balance between AI assistance and human care in mental health while ensuring transparency, accountability, informed consent, and accessibility to prevent negative consequences and promote ethical and responsible AI development and deployment.

**Potential benefits and challenges arising from the use of chatbots and artificial intelligence (AI)–enhanced internet-based platforms for the lesbian, gay, bisexual, transgender, and queer community.**

**Potential benefits**

- Accessible, fair, and inclusive support
- Anonymity
- Availability around the clock
- Tailored and relevant content
- Reduced isolation
- Access to accurate, evidence-based information
- Cultural sensitivity and competence
- Resource navigation

**Potential challenges**

- AI as a “black box”
- Biases affecting AI models, training data sets, and training procedures
- Algorithmic biases and misinterpretation
- Human error
- Legal and ethical concerns, including confidentiality and privacy
- Digital divide
- Lack of empathy and emotional support (“depersonalization of support”)
- Dependency (vs empowerment)

To the best of the authors’ knowledge, there is no comprehensive appraisal of the impacts of generative conversational AI on the LGBTQ community. Therefore, this review was conducted to fill this knowledge gap—the implications of its findings are practical, varied, and broad. AI is increasingly affecting various aspects of people’s lives worldwide, from everyday technology to advanced systems capable of autonomous decision-making. As previously mentioned, although AI holds significant promise, its potential risks depend on its design and application as AI technology can promote either equity and inclusion or exclusion among diverse societal groups. Understanding how AI is designed in terms of data selection, training, use, and integration into society is paramount to avoid harmful consequences such as fostering discrimination based on age, gender, sexuality, race, political beliefs, religious creed, or ability. Critically assessing the impact of AI on diverse communities and intentionally considering diversity-aware and intersectional approaches can contribute to shaping AI’s impact positively for society at large.

## Methods

### Study Design

This study was designed as a scoping review [13]. The 5-stage method by Arksey and O’Malley [14] was leveraged building on the recommendations proposed by Levac et al [15], who have expanded the methodology to comprise 6 or 7 stages. In stage 1, we formulated and refined the research question. In stage 2, we balanced feasibility and comprehensiveness. In stage 3, we adopted a team-based approach for study selection. In stage 4, we performed data extraction. In stage 5, we incorporated both quantitative and qualitative evidence using mixed methods analyses; we reported a range of relevant outcomes; and we considered implications for policy, practice, and future research. In stage 6, we integrated stakeholder consultation as an integral part of our knowledge translation and dissemination project. In stage 7, we provided suggestions and methodological guidance for future research on generative conversational AI for the LGBTQ community.

The findings are reported in accordance with the recommendations formulated in the PRISMA-ScR (Preferred Reporting Items for Systematic Reviews and Meta-Analyses extension for Scoping Reviews) checklist (Multimedia Appendix 1 [16]).

### Stage 1: Formulation of the Research Question

Our research question was as follows: “What is the impact of generative conversational AI on the LGBTQ community in terms of beneficial effects, potential, and opportunities as well as the detrimental effects, challenges, and threats?”

### Stage 2: Balancing Feasibility and Comprehensiveness

We assembled a multidisciplinary team comprising an expert in epidemiology, biostatistics and data science, and research and evidence synthesis methodologies; an expert in affirming psychotherapy; an expert in LGBTQ psychiatry and mental health; an expert in pediatric neuropsychiatry; and an expert in sex and gender medicine. Of note, our team comprises members of the LGBTQ community.

### Stage 3: Literature Search

A total of 4 major scholarly databases were searched from inception: Scopus, Embase, Web of Science, and MEDLINE via its freely available PubMed interface. Moreover, gray literature (Google Scholar), search engines, and internet-based assistant web references were consulted as well.

The search string consisted of 2 major components: one part comprised keywords related to generative conversational AI and the other was related to the LGBTQ community. The full string is reported in Textbox 2 and in Multimedia Appendix 2. Medical Subject Heading (MeSH) terms and wildcard options were used where appropriate.

**Search strategy adopted in this scoping review.**

**Databases searched**

- PubMed or MEDLINE, Scopus, Web of Science, Embase, Google Scholar, search engines (Google), and internet-based assistant web references

**Search string**

- (chatbot* OR “virtual assistant*” OR “virtual agent*” OR “artificial intelligence” OR “generative AI” OR “conversational AI” OR “large language model*” OR LLM OR LLMs OR ChatGPT OR Bard) AND (LGBT* OR homosexual* OR queer OR lesbian* OR bisexual* OR pansexual* OR intersex* OR transgender OR transsexual* OR “men who have sex with men” OR “men having sex with men” OR “MSM community” OR genderfluid OR genderqueer OR gender-diverse OR gender-expanded OR “gender non-conforming” OR nonbinary OR “sexual and gender minorities” OR “sexual minority” OR “gender minority” OR “sexual orientation” OR “sexual identity” OR “gender identity” OR “gender expression” OR sexuality)

**Inclusion criteria**

- Population: the lesbian, gay, bisexual, transgender, and queer (LGBTQ) community and counselors working with this community
- Intervention or exposure: exposed to chatbots and artificial intelligence (AI)–enhanced internet-based platforms
- Comparator or comparison: any comparator or comparison (chatbot and AI-enhanced internet-based platform vs in-person support, live real-time chat with a human, semiautomated devices, mobile-based apps, sex diaries, and other modalities)
- Outcomes: feasibility, acceptance, and effectiveness of the chatbot or AI-enhanced internet-based platform
- Study design: peer-reviewed original research of any design (observational, interventional, cross-sectional, longitudinal, nonrandomized, and randomized controlled trial)

**Exclusion criteria**

- Population: populations other than the LGBTQ community and counselors working with this community
- Intervention or exposure: exposed to in-person support, live real-time chat with a human, semiautomated devices, mobile-based apps, sex diaries, and other modalities
- Comparator or comparison: not applicable
- Outcomes: acceptance or acceptability only or outcomes other than the effectiveness of the chatbot or AI-enhanced internet-based platform
- Study design: editorials, letters to the editor, commentaries, expert opinions, clinical cases and reports, and reviews (of any type)

**Time filter**

- From inception

**Language restrictions**

- None applied

**Target journals**

- *Digital Culture & Education, Frontiers in Digital Health, Internet Intervention, JMIR Formative Research, JMIR Research Protocols, JMIR,* and *Sexual Health*

The search string was formulated after an initial familiarization with the extant literature and refined with the help of an expert librarian, an expert in research and evidence synthesis methodologies, and stakeholders from the LGBTQ community.

The inclusion or exclusion criteria were formulated according to the Population, Intervention, Comparator, Outcome, and Study Design or Population, Exposure, Comparator, Outcome, and Study Design frameworks. They are shown in Textbox 2 along with other details. The population was the LGBTQ community and professionals working with its members, whereas studies recruiting other populations were excluded.

Intervention or exposure was the exposure to chatbots and AI-enhanced internet-based platforms. It is important to stress that this scoping review covered interventions delivered by means of conversational agents. Compared with static smartphone apps, they can be deployed within social media sites, not requiring user download and not taking up space in users’ devices. Interventions administered via web, social media, or mobile apps not involving conversational agents or chatbots but “relational” or “embodied agents” with limited interactivity were excluded. Furthermore, there exist first-generation chatbots, also known as rule-based chatbots, that are limited to predefined scripts, whereas the next generation of generative conversational AI systems exhibits the groundbreaking characteristic of so-called “open-domain conversations,” being able to engage in open-domain conversations on a variety of topics. This flexibility allows for more dynamic, on-demand, customized, and immersive interactions. To do so, generative conversational AI often uses machine learning techniques to learn from vast amounts of text data, capture language patterns, and generate humanlike responses. Both generations of chatbots were included in this scoping review.

Comparator or comparison was any comparator or comparison, including chatbot and AI-enhanced internet-based platform versus in-person support, live real-time chat with a human, semiautomated devices, mobile-based apps, sex diaries, or other delivery modalities.

Outcome included a range of outcomes encompassing feasibility, acceptance, and effectiveness of the intervention delivered by chatbots or AI-enhanced internet-based platforms. Studies on acceptance or acceptability only or other outcomes different from the effectiveness of the chatbot or AI-enhanced internet-based platform were not deemed eligible for inclusion in this scoping review.

Finally, study design was any peer-reviewed original research designed as an observational, interventional, cross-sectional, longitudinal, nonrandomized, or randomized controlled trial (RCT). Studies designed as editorials, letters to the editor, commentaries, expert opinions, clinical cases and reports, or reviews were not retained in this scoping review.

The literature search was conducted from inception, without language restrictions, and was updated as of September 27, 2023.

### Stage 4: Data Extraction

In total, 2 authors independently extracted the relevant data related to the first author of the study, year of publication, study country, study design, name of the application, and its aim and target users. A specifically designed Excel (Microsoft Corp) spreadsheet was used for data abstraction and tabulation. Disagreements between the 2 authors (if any) were resolved through discussion during the data extraction process. Overall, interrater reliability was excellent (*k*>0.90).

### Stage 5: Research Implications

We examined the potential consequences of our findings on policy development, practical implementation, and the direction of future research endeavors. We assessed whether and how our research findings aligned with existing regulations and considered how this would imply changes in policies and the implementation of novel guidelines. In addition, we identified practical applications of our research findings in real-world settings and any challenges or barriers to their implementation. We considered the broader societal impact of our findings and analyzed both short- and long-term effects on LGBTQ individuals and the LGBTQ community at large.

### Stage 6: Stakeholders’ Involvement and Engagement

We integrated vital components in our research by involving stakeholders in the process, thereby making stakeholder consultation an integral part of our efforts to translate and disseminate knowledge effectively. We identified key stakeholders (mainly LGBTQ organizations) relevant to our research findings and reached out to them to engage them by presenting and discussing the study idea, protocol, and preliminary findings. We also organized workshops, seminars or webinars, and briefings to facilitate dialogue and make the conversation more interactive. Through participatory research, we gathered their feedback, which was implemented accordingly during the research and drafting of this manuscript. More specifically, stakeholders formulated a checklist of features and parameters that they considered valuable during AI development and deployment within the LGBTQ community: user experience feedback, content relevance, bias detection, privacy concerns, ethical concerns, cultural sensitivity and competence, impact assessment, intersectionality considerations, and community-centered approach.

### Stage 7: Recommendations for Future Research

After mapping the state of the art, we identified gaps in knowledge, unanswered questions, and emerging issues. We outlined the key areas that should be explored in future research, and based on this, we offered recommendations and practical guidance for forthcoming research endeavors centered on the use of generative conversational AI in the context of the LGBTQ community. Our aim was to provide practical, valuable insights that could inform the design, execution, and evaluation of studies in this domain, thereby contributing to the enhancement of generative conversational AI’s applicability and effectiveness for addressing the unique needs and challenges faced by the LGBTQ community.

## Results

### Search Results

The initial literature search yielded 1390 items: 439 (31.58%) from Embase, 333 (23.96%) from MEDLINE or PubMed, 312 (22.45%) from Scopus, and 306 (22.01%) from Web of Science. After removing 15.04% (209/1390) of duplicate articles, 1181 items were screened for eligibility. A total of 3 additional items were identified through a search of the gray literature. After removing 98.23% (1163/1184) of irrelevant items, 21 studies were examined in depth. Of these 21 studies, 12 (57%) [17-28] were excluded with reasons (Textbox 3): 3 (25%) [21,23,25] were excluded as they were chatbot acceptance studies only without implementation of the chatbot project; 3 (25%) [17,18,22] were excluded as they described digital tools, which, despite being AI-enhanced, did not fully meet our inclusion criteria (not being able to dynamically interact with the client or patient); 1 (8%) [17] was a usability study that lacked the implementation part; 1 (8%) [19] was not retained as it performed a content analysis of health-related information concerning HIV pre-exposure prophylaxis (PrEP); another study (8%) [20] was excluded as it explored how text-based conversational agents express their gender and sexual identity, which was not related to our topic of interest and research aims or objectives; and 3 (25%) further studies [24,27,28] were excluded as the population also comprised non-LGBTQ individuals. Finally, an app called Rainbow Austin [26] was excluded because of lack of details.

**Articles excluded and the reasons for exclusion.**

**Biernesser et al [17], 2023**

- Intervention or exposure: exposure to a “partially automated, SMS text message intervention leveraging web-based content” and not to a chatbot
- Outcomes: usability analysis

**Buchbinder et al [18], 2023**

- Intervention or exposure: a static digital tool (an app) and not a dynamic, interactive digital tool (a chatbot)

**Darien et al [19], 2023**

- Outcomes: content analysis of health-related information (specifically concerning pre-exposure prophylaxis)

**Edwards et al [20], 2021**

- Outcomes: how conversational agents express their gender and sexual identity

**Henkel et al [21], 2023**

- Outcomes: acceptability of chatbots and artificial intelligence (AI)–enhanced virtual platforms

**Liu et al [22], 2021**

- Intervention or exposure: an app and not a chatbot

**Millman et al [23], 2023**

- Outcomes: acceptability of chatbots and AI-enhanced internet-based platforms

**Nadarzynski et al [24], 2021**

- Population: population comprising also non–lesbian, gay, bisexual, transgender, and queer (LGBTQ) individuals

**Peng et al [25], 2022**

- Outcomes: acceptability of chatbots and AI-enhanced internet-based platforms

**Cantu [26]**

- Lack of details

**Sanabria et al [27], 2023**

- Population: population comprising also non-LGBTQ individuals

**Wang et al [28], 2022**

- Population: population comprising also non-LGBTQ individuals

### Description of the AI Tools Identified

**Figure 1:**
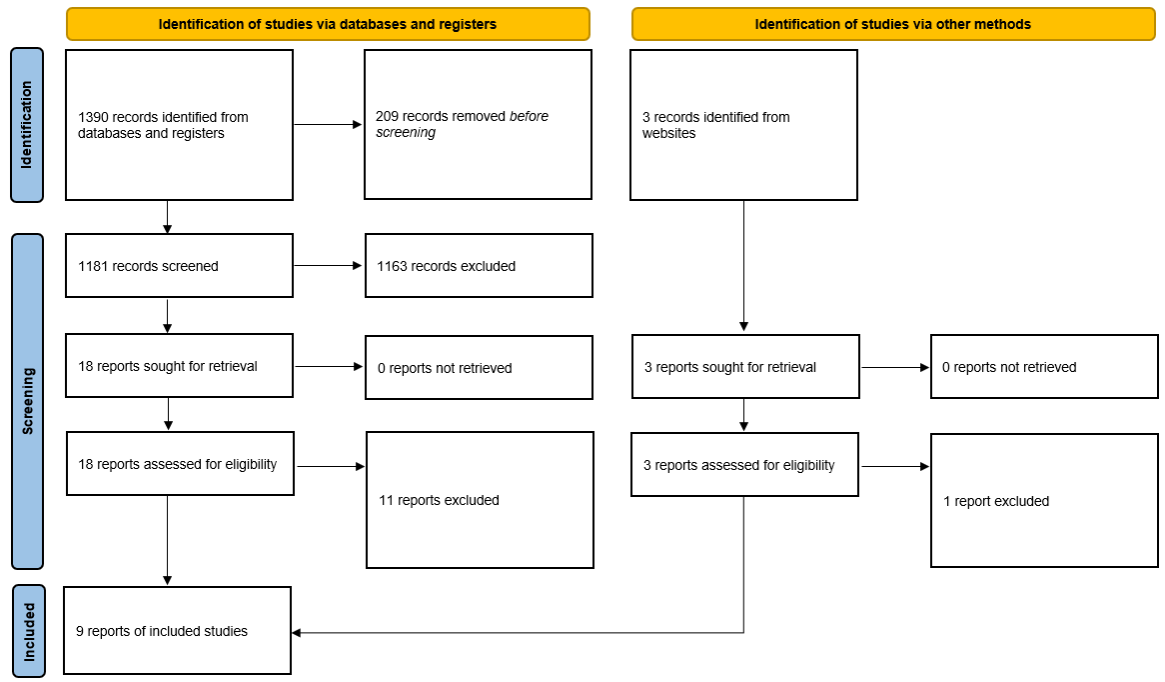
Pictorial flowchart used in this scoping review.

A total of 9 studies [29-37] were retained in this scoping review. Overall, also including web resources, 7 AI-enhanced applications (Amanda Selfie [33], Crisis Contact Simulator [37], HIVST-Chatbot [30], Queer AI [36], REALbot [31,32], TelePrEP Navigator [29], and Tough Talks [34,35]) were reviewed in this study (Figure 1 and Table 1).

The chatbots and internet-based assistants identified served various purposes: (1) to identify LGBTQ individuals at risk of suicide or contracting HIV or other sexually transmitted infections, (2) to provide resources to LGBTQ youth from underserved areas, (3) to facilitate HIV status disclosure to sex partners, and (4) to develop training role-play personas encompassing the diverse experiences and intersecting identities of LGBTQ youth and educate counselors.

**Table 1:** Chatbots targeting the lesbian, gay, bisexual, transgender, and queer (LGBTQ) community and professionals working with its members and related studies included in this scoping review.

Application name	Study	Study design	Country	Programming language or languages	Platform	Theoretical framework	Aim	Target users	Development and implementation
Amanda Selfie	Massa et al [33]	Formative study embedded in an RCT^a^	Salvador, Belo Horizonte, and São Paulo (Brazil)	Dialogflow as AI^b^ engine using Perl and JavaScript with Bottender as development framework communicating with the application programming interface via the Catalyst and Mojo frameworks and with the trial database via PHP^c^	Facebook Messenger	LGBTQ theories	HIV PrEP^d^ enrollment, linkage, and retention	Members of the LGBTQ community at risk of contracting HIV (young MSM^e^ and transgender women or girls aged 15-19 y)	3 stages (conception, trial, and final version)
Crisis Contact Simulator	Ruiz [37]	N/A^f^	United States	N/A	N/A	N/A	Crisis	Counselors of members of the LGBTQ community at risk of mental health issues	N/A
HIVST-Chatbot	Chen et al [30]	Protocol for a noninferiority RCT	Hong Kong	N/A	WhatsApp	N/A	HIV self-testing	Members of the LGBTQ community at risk of contracting HIV	N/A
Queer AI	Martinez [36]	N/A	United States	N/A	N/A	Feminist and LGBTQ theories	Community empowerment and connection	Members of the LGBTQ community	N/A
REALbot	Escoobar-Viera et al [31,32]	1-wk exploratory pilot study with a single-group, pretest-posttest design	United States	N/A	Facebook Messenger and Instagram	N/A	Enhancing the social media skills, reducing feelings of isolation, and strengthening connections among LGBTQ youth living in underserved, rural areas	Members of the LGBTQ community	Development, acceptability, usability, satisfaction, and utility testing
TelePrEP Navigator	Braddock et al [29]	Protocol for a trial	Louisiana, United States	Twilio, Studio, and Autopilot	SMS text messaging	N/A	HIV PrEP enrollment	Members of the LGBTQ community at risk of contracting HIV	2 stages (conceptualization and iterative community-engaged development process through 5 rounds of review and feedback)
Tough Talks	Muessig et al [34] and Hightow-Wiedman et al [35]	Formative study	United States	N/A	Mobile device	Social cognitive theory	HIV status disclosure	Young members of the LGBTQ community living with HIV	5 stages

^a^RCT: randomized controlled trial.

^b^AI: artificial intelligence.

^c^PHP: Hypertext Preprocessor.

^d^PrEP: pre-exposure prophylaxis.

^e^MSM: men who have sex with men.

^f^N/A: not applicable.

### AI Design, Development, User Feedback, and Results

#### Amanda Selfie

Amanda Selfie [33] is an empowering and intersecting avatar “persona” self-identifying as a Black transgender woman who uses a gender-neutral version of the Pajubá language to communicate. Pajubá is a creole language rooted in Portuguese that emerged through the integration of lexicons from Yorubá, Nagô, and various other African languages, which began to be used by the Brazilian LGBTQ community during the military government of Brazil (1964-1985) to escape police repression as homosexuality was criminalized. Amanda Selfie was designed with the intention of providing information regarding sexual health, in particular HIV, and matters related to gender identity or expression. More in detail, this AI-enhanced platform was created to screen young adults who self-identify as men who have sex with men (MSM) and transgender adolescent girls and young women aged 15 to 19 years and identify those at risk in terms of HIV infection susceptibility through the administration of a web-based questionnaire. The platform also facilitates the scheduling of appointments for the PrEP1519 study—an RCT in which the formative study describing Amanda Selfie is embedded—in particular for HIV PrEP enrollment. Specifically, Amanda Selfie aimed to enhance the connection with and retention of adolescents within PrEP clinics by identifying situations posing a risk that necessitated clinical care for sexually transmitted infections and postexposure prophylaxis. In addition to assisting in making appointments as per user requests, the platform delivered reminders for PrEP pill consumption and even provided HIV self-tests sent through mail. The chatbot was developed for the Facebook Messenger platform and used Dialogflow, an AI engine developed by Google LLC or Alphabet Inc. The programming languages used were Perl and JavaScript. The development framework Bottender was used, which communicated with a Perl-based application programming interface (API) using the Catalyst and Mojo frameworks. The choice of Facebook Messenger was motivated by its capacity to facilitate complex conversational flows and its offering of free mobile data packages in Brazil. The 3-stage formative study was conducted in 3 Brazilian capital cities: Salvador, Belo Horizonte, and São Paulo. The initial phase involved (1) the conception of the avatar persona (Amanda Selfie), their visual representation, and the language they would use; (2) the definition of goals; and (3) the design of both an open conversational flow and a closed flow designed to generate demand from the user. In addition, it included creating a monitoring panel for tracking interactions and integrating it with the RCT database. This segment occurred over 8 workshops that involved professionals and researchers from the fields of communication, health, and IT along with the LGBTQ community. Acceptability was assessed through 9 in-depth individual interviews with adolescents and 2 discussion groups each consisting of 6 peer educators. The second phase was the trial of the chatbot, and the third phase was the release of the final version of the product, which was revised based on feedback and comments provided by the testers. Quizzes were removed and more information was added, giving more time for the user to react. Some users emphasized the significance of transitioning to an in-person conversation with an actual individual when discussing subjects demanding more in-depth exploration and complained of the inaccuracy of some replies provided by Amanda Selfie. Finally, from a quantitative standpoint, Amanda Selfie contributed to a 2.4% increase in the number of PrEP uptake events.

In conclusion, Amanda Selfie is an AI-enhanced chatbot avatar designed through participatory co-design as a Black transgender woman to provide information on sexual health, especially HIV, and support gender identity or expression issues targeting young adults, specifically MSM and transgender girls; assessing HIV risk; scheduling PrEP appointments; offering reminders; and even providing HIV self-tests by mail. In a 3-stage study in Brazil, Amanda Selfie increased PrEP uptake events.

#### Crisis Contact Simulator

Since the beginning of 2021, the Trevor Project, the largest organization dedicated to preventing LGBTQ youth suicide, has used an AI technology called the Crisis Contact Simulator [37] to educate its counselors on effectively communicating with young individuals facing crisis situations. This tool essentially emulates potential conversations through AI-driven chatbots. Initially, the Crisis Contact Simulator introduced a persona named Riley and, subsequently, a second one named Drew; these chatbots embody fictional young persons in their early 20s residing in California dealing with bullying and harassment. This technology has assisted in training >1000 counselors. It is important to note that The Trevor Project uses the Crisis Contact Simulator to supplement its existing training methods and not to replace in-person training. This tool enables new trainees to engage in 2 role-play sessions at their convenience. In addition, the chatbots are designed to accurately reflect the language and communication style of today’s Generation Z youth, including the “realistic” use of capitalization and punctuation.

No further details are available concerning, for instance, the technical development, implementation, and clinical effectiveness of the chatbot.

In conclusion, since 2021, the Trevor Project has used the Crisis Contact Simulator, an AI tool, to train >1000 counselors in effective communication with young individuals facing crises. The Crisis Contact Simulator features chatbot personas, Riley and Drew, who simulate conversations with young adults dealing with bullying and aims to supplement, not replace, in-person training while authentically mirroring Generation Z communication styles.

#### HIVST-Chatbot

HIVST-Chatbot [30] is a chatbot developed in Hong Kong specifically targeting the MSM community. The WhatsApp platform via its public Web API services was leveraged for the deployment of the application. Participant messages can be transmitted to both WhatsApp’s instant messaging server and an independent chatbot system, which consists of an administrative system and the chatbot itself. This system processes the messages and promptly sends a response back to the WhatsApp server. The entire process occurs in a fraction of a second, ensuring a seamless user experience without any perceptible delay. The chatbot system comprises 3 key components: the dialogue management module, user management module, and multimedia management module. The first captures and maintains all user-chatbot conversations, extracting contextual data, including user activity statistics and previous interactions between the user and the chatbot. The natural language processing module scrutinizes the textual content of each message. Subsequently, the system directs the message to prompt actions based on a blend of techniques, including the use of predefined rules (the so-called rule-based interactions triggered by messages containing particular keywords) and the identification of conversation patterns (“learning-based interactions,” which enable the widening of the repertoire of responses, making them more appropriate and relevant). Specific “conversation templates” such as the persuasion template are gauged based on the ongoing stance of the conversation to continue the interaction or initiate new conversations switching to new topics. The user management module logs comprehensive details of user interactions. Administrators have the capability to associate users’ WhatsApp numbers with the chatbot and monitor the advancement of intervention delivery. This encompasses tracking metrics such as completed sessions and user-chatbot disconnections. Finally, the multimedia management module enables the uploading, sending, updating, and eventual exchange of images and videos. The chatbot was pretrained on a knowledge graph that connected questions (concerns about HIV, HIV testing, and HIV self-testing from 10 local MSM) and the corresponding responses provided by experienced HIV testing administrators, community-based organization personnel, and HIV prevention experts. The effectiveness of HIVST-Chatbot will be evaluated by means of a parallel-group, noninferiority RCT with a follow-up at 6 months, recruiting 528 participants and allocating 264 to the intervention with the chatbot and the remaining 264 to the HIV self-testing service with web-based real-time instruction and pre- and posttest counseling provided by trained administrators. The outcomes that will be collected and measured include uptake of HIV self-testing (primary outcome), rates of risky sexual behaviors—prevalence of condomless anal intercourse and multiple male sexual partners—and uptake of HIV testing other than self-testing (secondary outcomes).

In conclusion, the HIVST-Chatbot was developed in Hong Kong primarily targeting the MSM community. It operates on the WhatsApp platform using its public Web API services and swiftly processes user messages. The chatbot system consists of dialogue management, user management, and multimedia management modules, using natural language processing techniques and predefined rules to engage users in conversations about HIV and HIV testing. It was pretrained using knowledge from local experts and will be evaluated in an RCT involving 528 participants to assess its impact on HIV self-testing uptake and rates of risky sexual behaviors.

#### Queer AI

Queer AI [36] originated as a collaborative effort between Emily Martinez, a first-generation immigrant or refugee from Cuba relocated to Miami, Florida, and new media artist, and Ben Lerchin, an engineer, web developer, designer, and artist, in 2018. Their aim was to develop a conversational AI that captured the complexity of relationships, physicality, and personal identity based on erotic literature, feminist and LGBTQ theory, and ethics of embodiment. The initial version of Queer AI (version 1) was trained using >50,000 conversational exchanges sourced from scripts from LGBTQ theater using a recurrent neural network–based sequential-to-sequential approach. Lerchin was responsible for coding and data preparation, whereas Martinez authored manifestos and engaged with the chatbots. In the subsequent iteration (2020), they transitioned to using GPT-2, a robust transformer-based LLM with 1.5 billion parameters, which was trained on a data set containing 8 million web pages. During her involvement in the ML5 fellowship project, Martinez undertook the training of this model. Moreover, the project encompasses the creation of AI-generated zines and the facilitation of community workshops.

No further details are available concerning, for instance, the technical development, implementation, and clinical effectiveness of the chatbot.

In conclusion, Queer AI is a collaborative project initiated in 2018 that aimed to create a conversational AI system that captured the complexity of human sexuality in terms of relationships, physicality, and personal identity based on a complex theoretical framework and a unique blend of sources and conceptual references, from erotic literature to feminist and LGBTQ theory and embodiment ethics. Initially trained on 50,000 conversational exchanges from LGBTQ theater scripts using a recurrent neural network, it later transitioned to using the GPT-2 LLM with 1.5 billion parameters in 2020. Furthermore, the project involves creating AI-generated zines and conducting community workshops.

#### REALbot

REALbot [31,32] is an automated educational intervention devised with the help of 2 research assistants and a private hosting service through a human-centered design that operates through a rule chatbot on social media platforms such as Facebook Messenger and Instagram. It is aimed at enhancing social media skills, reducing feelings of isolation, and strengthening connections among LGBTQ youth living in underserved, rural areas. REALbot delivers a range of topics comprising a series of infographics, animated short videos, short text stories with alternate endings, and testimonies and suggestions from other rural-living LGBTQ youth that cover 4 main topics on target social media behaviors and interactions (ie, “avoiding negative content and interactions or reneging negativity,” “keeping a balanced engagement or engaging with balance,” “connecting with real and actual allies,” and “limiting use”). In a previous formative work [31], participants expressed a desire for various aspects, such as visibility and positive representation of LGBTQ youth individuals and gaining insights from those who share similar life experiences. They also indicated an interest in understanding the functionalities of social media platforms that enable them to connect with diverse audiences and enhance their online safety. Furthermore, young individuals favored using LGBTQ-specific communities within their existing social media accounts while maintaining a level of privacy regarding their personal information. After the formative work, the authors conducted a week-long exploratory study involving a group of 20 LGBTQ youth aged 14 to 20 years from rural areas out of an initial list of 348 survey respondents recruited via social media and gauged their social media self-efficacy, perceived isolation, and depressive symptoms through pre- and posttest assessments. Half of the participants self-identified as transgender, and 35% identified as cisgender gay men or lesbians. They were mostly White (75%), living with parents or guardians (90%), and in high school (80%). After the intervention, the authors examined the chatbot’s acceptability, usability, and satisfaction. In addition, they gathered feedback from participants on their preferences and dislikes regarding the intervention in response to 2 open-ended questions, which were analyzed using content analysis. REALbot demonstrated a high level of acceptability and received favorable usability ratings. Feedback from participants was categorized into 2 main themes: usability and the content provided in relation to sexuality and various components of social life. User satisfaction with the chatbot was also high even though a few participants complained that the chatbot, despite being friendly, welcoming, and respectful, covered limited content, appeared mechanical, and had constraints in its capacity to recall and incorporate information previously provided by the user into a seamless conversation during subsequent interactions. Participants engaged actively with the chatbot. However, there were no statistically significant changes in pre- and posttest scores for most of the outcomes, and the effect sizes were generally modest, with Cohen *d* values ranging from −0.36 to 0.77, being small for social isolation, depressive symptoms, and overall social media skills and medium for ability to find content on social media.

In conclusion, REALbot is an automated educational intervention specifically designed for rural LGBTQ youth to improve their social media skills and reduce isolation. Although it received high user satisfaction and usability ratings, the intervention had a limited impact on measured outcomes, suggesting the need for further refinement and content enhancement to achieve its intended goals.

#### TelePrEP Navigator

TelePrEP Navigator [29], a rule-based chatbot, was developed through an iterative, community-engaged process. It is an SMS text messaging–based chatbot tailored to LGBTQ adolescents and young adults. It allows for participating in discussions concerning PrEP eligibility, aiding in the enrollment process, scheduling appointments, identifying and resolving barriers to accessing PrEP (such as insurance-related concerns), and furnishing answers to frequently asked questions. The pilot consisted of 2 phases: phase 1 (conceptualization) and phase 2 (development process through 5 rounds of review and feedback). During the pilot test, developers and behavioral researchers learned to avoid sending too many redundant messages asking unnecessary questions and containing links to external resources. Concise, relevant text was more appreciated by testers. Some testers complained of inaccurate replies provided too quickly.

No further details are available concerning, for instance, the technical development of the chatbot.

In conclusion, the TelePrEP Navigator is an SMS text messaging–based chatbot designed for LGBTQ adolescents and young adults that focuses on PrEP. It helps with PrEP eligibility discussions, enrollment, appointment scheduling, addressing access barriers such as insurance issues, and answering frequently asked questions. The development process involved iterative community engagement and 2 pilot phases. Feedback from testers highlighted the importance of concise, relevant messages but also pointed out the need for more accurate and thoughtful responses as some answers were provided too quickly.

#### Tough Talks

Tough Talks [34,35] is an AI-enhanced, multicomponent, graphically appealing mobile health intervention that facilitates role-playing scenarios and HIV status disclosure to intimate (romantic or sexual) partners, friends, and family members. Content addresses cognitive (HIV-related literacy), normative (legal and ethical implications of one’s HIV status and failure to disclose it), educational (awareness of HIV and safer sex), experiential (previous negative experiences with HIV status disclosure), and emotional (HIV-related stigma and fear of rejection) aspects. Environmental (intersectional stigma, social norms, and regional context) and behavioral (attitudes, practices, and communication skills) factors are also addressed. Tough Talks was developed in 2019 as a result of a partnership between the University of North Carolina at Chapel Hill (United States) and Virtually Better, Inc, bringing together specialists from diverse disciplines spanning HIV prevention, clinical psychology, computer science, and product development. Throughout the formative process, input from the MSM community was actively sought to guarantee that the app’s content, context, and functionality were pertinent and well received. The process and outcomes of devising this comprehensive, theory-driven intervention are outlined across various stages. A stand-alone interactive dialogue component consisting of various modules—introduction, module 1 (“Understanding disclosure”), module 2 (“Should I disclose?”), module 3 (“How do I disclose”), and module 4 (“Preparing for the outcome”)—was conceptualized and honed throughout phases 1 to 3. Phase 1 involved participants from 4 focus groups (n=58) who shared their previous experiences of disclosure, deliberated on strategies for disclosure (both challenges and facilitators), collaboratively devised real-life disclosure scenarios, and contributed to the creation of the stand-alone interactive dialogue feature. During phase 2, the dialogue feature underwent further refinement through 3 rounds of usability testing with youth from the MSM community living with HIV (n=32). In phase 3, the feature underwent preliminary efficacy testing with 11 new young MSM living with HIV. The Tough Talks prototype, as it stood at the conclusion of phase 3, featured an interactive internet-based dialogue component informed by social cognitive theory. This component guided users through conversations about disclosing their HIV status. In phase 4, the fully integrated intervention was developed through a mobile app, whereas phase 5 concerned the usability testing process (n=8 testers sampled from a clinical setting; mean age of 27.6 years; all self-identifying as Black; mean time since HIV diagnosis of 4.6 years; range 5 months-12 years; virally suppressed, with self-reported ≥90% antiretroviral therapy adherence in the previous month). All participants were in care, virally suppressed, and self-reported ≥90% antiretroviral therapy adherence in the previous month. In phase 4, disclosure dialogues were collected through web-based crowdsourcing in the form of a comic book contest, with 8 comic book panels. In addition to the modular components, Tough Talks has a back-end feature, a reporting system able to track user intervention analytics (metadata or paradata, such as the number of activities or tasks completed, number of log-ins, and time spent on the app), and an administrator portal to move the tool toward clinical testing. The app relies on an “utterance database,” and the avatar reacts (positively [showing support], negatively [lacking support], or neutrally) according to the type of scenario chosen, the conversation’s tone, user input, and other contextual factors. On the basis of feedback provided by the 8 testers in phase 5, some changes were implemented. The most notable enhancements proposed were focused on the AI technology itself. Incorporating speech recognition was a key addition, eliminating the need for participants to type their responses. Revisions were also made to address emotional considerations—the coach’s dialogue was bolstered to provide more effective guidance through the scenario; reflection activities were introduced following disclosure scenarios to help users process the information they received during the scenario; and the availability of internet-based and in-person support was confirmed, encompassing local and web-based resources for sexual and mental health as well as contact information for staff and clinics. Finally, the authors have planned a multisite RCT aimed at exploring and comparing the acceptability and effectiveness of the AI (fully automated) and Wizard of Oz (semiautomated) supported dialogue platform.

In conclusion, Tough Talks is a mobile health intervention developed through a partnership between the University of North Carolina at Chapel Hill and Virtually Better, Inc. It helps individuals role-play and navigate HIV disclosure conversations with intimate partners, friends, and family. The intervention, delivered as a mobile app, incorporates AI technology, crowdsourced dialogue scenarios, and usability testing to enhance the user experience, and there are plans to conduct an RCT to assess its acceptability and effectiveness.

## Discussion

### Opportunities of AI for the LGBTQ Community

AI has the potential to both positively and negatively affect the LGBTQ community [38]. Positive impacts can encompass inclusion and diversity. AI-driven technologies help increase the representation and visibility of LGBTQ individuals in media, entertainment, and other content, where they are usually underrepresented. AI algorithms can be trained to recognize and promote diverse voices and stories, contributing to a more inclusive narrative. In particular, the positive and authentic representation of multiple, intersecting identities (as in the case of the chatbot persona of Amanda Selfie) validates the existence and experiences of LGBTQ individuals, acknowledging that they are an integral part of society; promotes a sense of belonging and inclusivity helping combat feelings of isolation and marginalization that LGBTQ individuals may experience; fosters understanding and awareness of diverse forms of sexuality, reducing stereotypes and prejudice; and has a positive impact on mental health, boosting self-esteem and reducing mental health disparities.

As such, AI could promote more supportive communities. AI-powered platforms and chatbots provide a safer space for LGBTQ individuals to connect, seek support, and discuss their experiences. These platforms, for instance, Tough Talks, help combat feelings of isolation and provide access to resources and information and a sense of community.

In particular, they provide valuable mental health support. AI-driven mental health apps and chatbots offer personalized support for LGBTQ individuals struggling with mental health challenges [39,40]. These tools provide resources, coping strategies, and even crisis intervention, potentially helping reduce mental health disparities. They can enhance sexual health and promote positive behavior changes [41-43] through a variety of strategies, including goal setting, monitoring, real-time reinforcement or feedback, and on-demand support. All this can lead to health care advancements. AI aids in medical research and health care by identifying patterns and trends related to health disparities experienced by LGBTQ individuals. This could lead to better informed medical practices and interventions tailored to the unique needs of the LGBTQ community. In some of the studies reviewed, preliminary findings confirmed the usefulness of AI-enhanced chatbots in favoring positive behavior changes, such as in the case of Amanda Selfie, where an increase in the number of PrEP uptake events was reported.

Moreover, AI-enhanced devices offer a variety of services, including language translation to promote more inclusivity worldwide. AI-powered language translation tools can facilitate communication across diverse languages, promoting inclusivity and understanding among the global LGBTQ community. AI-enhanced tools educate, empower, and advocate. AI-driven educational platforms can disseminate accurate information about human sexuality in terms of sexual orientation and gender identity or expression, helping combat and reduce misinformation, stereotypes, and stigma.

### Challenges and Concerns of AI for the LGBTQ Community

However, there are challenges and concerns as well, such as bias and discrimination. AI algorithms can inadvertently perpetuate biases present in the data they are trained on. If the training data contain biases, AI systems may reproduce discriminatory or harmful behaviors, exacerbating existing challenges faced by the LGBTQ community. As stated by Seaborn et al [44], it is important to pay attention to “explicit biases in language, especially against women, girls, femme-identifying people, and genderqueer folk; implicit associations through word embeddings; and limited models of gender and masculinities, especially toxic masculinities, conflation of sex and gender, and a sex/gender binary framing of the masculine as diametric to the feminine.” Data sets used to train chatbots and internet-based assistants may contain biases, and this could, in turn, magnify gender-related biases [45-49]. A solution to these issues could be using inclusive data sets, as in the case of Queer AI, which makes use of conversational exchanges sourced from scripts from LGBTQ theater and feminist literature.

Other issues are privacy concerns. LGBTQ individuals might be concerned about the potential misuse of AI-generated information related to their sexuality. More specifically, members of the LGBTQ community may worry about the possible exploitation of AI-generated data pertaining to their sexual orientation or gender identity or expression. These concerns become especially prominent in areas with limited legal safeguards in place. There is also the alarming possibility of individuals using AI and other web-based tools to locate and harass members of the LGBTQ community, thereby exacerbating the misuse of such technology to target and intimidate individuals based on their sexual orientation or gender identity or expression. This underscores the pressing need for robust protections and responsible AI use to safeguard the rights and safety of LGBTQ individuals in digital spaces [50].

Another crucial issue is dating apps and safety. Although AI can enhance the matching algorithms in dating apps, it is important to consider potential safety concerns as some LGBTQ individuals might be vulnerable to harassment or violence when their identities are disclosed on such platforms. There are also ethical dilemmas. The development of AI technologies that can manipulate images or videos could lead to new forms of harassment or “outing” of individuals who are not publicly out about their sexual orientation or “misgendering” them concerning their gender identity or expression. Identity recognition is another concern. Facial recognition technology could raise concerns about outing or misgendering transgender or intersex individuals without their consent or compromising their safety. Finally, access to AI should be equitable. Technological advancements can sometimes exacerbate existing inequalities. LGBTQ individuals with limited access to technology or digital literacy might be left behind in terms of benefiting from positive AI impacts [51-53].

### Generative Conversational AI for the LGBTQ Community: State of the Art and Future Directions

The studies in this review show that the use of generative conversational AI for the LGBTQ community is still in its infancy. Generally, the deployment of chatbots was deemed both feasible and well received, with strong ratings for usability and user satisfaction. Room for improvement concerning the content offered and more engaging, interactive conversations was identified. Moreover, the studies included in this scoping review used small sample sizes and deployed interventions of short duration, measuring a limited number of outcomes (both in terms of psychological constructs and objective outcome measures). As such, further development and a formal evaluation of engagement with behavioral objectives; high-quality, randomized interventions and trials comparing different content and topics, delivery modalities (such as delivery via peer and community support or counseling), and availability of platforms for dissemination; and implementation of interventions of longer duration are warranted.

In summary, AI has the potential to contribute positively to the LGBTQ community by providing representation, support, and resources. However, it is crucial to address biases and privacy, legal, and ethical concerns to ensure that AI technologies do not perpetuate harm or discrimination against the community. Developers, policy makers, LGBTQ advocates, allies, and other relevant stakeholders need to work together to create AI systems that are inclusive, fair, and respectful of the rights and well-being of LGBTQ individuals.

### Recommendations

On the basis of the findings of our scoping review, we would like to provide actionable steps through which developers, policy makers, and other relevant stakeholders can collectively work toward the responsible and ethical development and deployment of AI-enhanced tools and platforms for the LGBTQ community, ensuring that they enhance the lives of LGBTQ individuals while minimizing harm and discrimination.

Chatbot developers should (1) assemble diverse development teams that include LGBTQ individuals to gain valuable insights and perspectives; (2) ensure that the data sets used for training AI models are diverse and representative and do not reinforce stereotypes or biases; (3) identify, adopt, and implement appropriate and rigorous bias detection and mitigation strategies during AI model development, testing, and deployment; (4) document AI algorithms, decision-making processes, and data sources transparently to build trust and facilitate accountability; and (5) actively seek and incorporate feedback from LGBTQ users to improve the AI-based tool. Furthermore, they should (6) prioritize strong data privacy measures given the sensitive nature of information related to sexual identity, sexual orientation, gender identity, and gender expression. It is also highly recommended that the chatbot development team is (7) culturally sensitive, competent, and trained in LGBTQ-related issues and terminology to avoid inadvertently causing harm or offense.

Concerning policy makers, they should (1) develop and implement regulations specifically addressing the ethical use of AI tools related to the LGBTQ community; (2) enforce transparency in AI development, with mandatory disclosures of data sources, decision-making processes, and bias mitigation efforts; and (3) establish mechanisms for holding developers accountable for any discriminatory or harmful outcomes of their AI tools. Moreover, they should (4) ensure that LGBTQ individuals have the same privacy and nondiscrimination protections in the digital space as they do in the physical world and (5) support the development of inclusive AI solutions.

Relevant stakeholders should (1) advocate for ethical AI development and deployment on behalf of the LGBTQ community, raising awareness about potential issues and challenges; (2) educate LGBTQ individuals about their rights and privacy and how to protect themselves when using AI-powered services; (3) support innovation by encouraging and promoting initiatives that aim to create AI tools and technologies that benefit the LGBTQ community; and (4) foster and fund research that examines the impact of AI on the LGBTQ community. Moreover, they should (5) monitor the use of AI in contexts relevant to the LGBTQ community and report instances of discrimination or harm. Finally, they should (6) promote collaboration among developers, policy makers, advocacy groups, and LGBTQ communities to ensure that AI solutions genuinely reflect and empower the community.

In conclusion, every single line and bit of code, every algorithm, and every data set used in AI systems must be scrutinized for biases and prejudices, and developers and policy makers should strive for a standard of AI that champions fairness and equality. The creation and deployment of AI technologies should be guided and informed by accountability and transparency, prioritizing the involvement of LGBTQ individuals in the development and evaluation of AI solutions (according to the principles of “responsible, intentional, and participatory co-design”). The lived experiences and perspectives of members of the LGBTQ community are, indeed, invaluable in ensuring that these technologies truly reflect their needs and aspirations (Textbox 4).

**Practical recommendations and actionable steps for each key stakeholder (chat development team members, policy makers, and other relevant stakeholders) in the development and deployment of artificial intelligence–enhanced solutions for the lesbian, gay, bisexual, transgender, and queer community.**

**Chat development team**

- Assembling of a diverse team
- Inclusive data
- Bias mitigation
- Transparency
- User feedback
- Privacy protection
- Cultural sensitivity and competence

**Policymakers**

- Regulation
- Transparency requirements
- Accountability
- User rights

**Other relevant stakeholders**

- Advocacy
- User education
- Innovation support
- Fundraising and research promotion
- Monitoring and reporting (“watchdog role”)
- Collaboration

### Strengths and Limitations

Our scoping review has a number of strengths, including its methodological rigor, compliance with high conducting and reporting standards, and reproducibility. In contrast, it suffers from some limitations that should be properly acknowledged. These include the relatively small number of studies that could be identified and summarized. In addition, a limited number of databases were explored. Some repositories that could be consulted in follow-up review studies and could provide additional insights include EBSCO, CINAHL, and APA PsycINFO.

### Conclusions

This study conducted a scoping review to investigate the impact of generative conversational AI on the LGBTQ community. In total, 7 applications, including chatbots and internet-based assistants, were identified and examined. These applications served various purposes, such as identifying at-risk LGBTQ individuals, providing resources to underserved LGBTQ youth, facilitating HIV status disclosure, and creating diverse training personas for counselors.

Leveraging chatbots and generative conversational AI can help address some of the unique challenges faced by the LGBTQ community, providing a safer, supportive, informed, nonjudgmental, internet-based environment where individuals can connect, seek guidance, and empower themselves. However, although generative conversational AI has significant potential, there are challenges to consider, such as the potential for generating inappropriate or biased content, the need for ongoing training and monitoring, and the difficulty in achieving truly humanlike responses consistently. Mental health professionals, members of the LGBTQ community, and other stakeholders, along with developers, are continually working to improve the technology’s accuracy, safety, and ethical use to ensure positive user experiences.

Interdisciplinary efforts and cooperative engagement among all stakeholders are essential to develop and deploy AI solutions that not only meet the technical requirements but also align with ethical values, respect individual rights, and positively affect the lives of the LGBTQ community. This collective approach is crucial for responsible and inclusive AI development.

In a world in which AI-enhanced platforms and technologies are shaping the human future at an unprecedented pace, the LGBTQ community stands at a crucial crossroads—the power of AI to promote and foster inclusivity, combat stigma, counter discrimination, and amplify LGBTQ voices is immense, but there are also the risks and dangers of perpetuating biases and magnifying stereotypes and harmful prejudices. It is a societal onus to commit to an ethical use of AI-based technologies in such a way that they are not only harnessed for innovation but also effectively, responsibly, and sensitively applied in all contexts relevant to the LGBTQ community for the sake of societal justice, fairness, and equity. Only in this way can AI become a force for social good, a beacon of hope, and an instrument of positive change for all regardless of one’s age, sex or gender, sexual orientation, gender identity or expression, race or ethnicity, political beliefs, religious creed, or ability.

## Appendix

Multimedia Appendix 1 Completed PRISMA-ScR (Preferred Reporting Items for Systematic Reviews and Meta-Analyses extension for Scoping Reviews) checklist.

Multimedia Appendix 2 Detailed search strategy for each database used in this scoping review.

## Abbreviations

AI: artificial intelligence
API: application programming interface
LGBTQ: lesbian, gay, bisexual, transgender, and queer
LLM: large language model
MeSH: Medical Subject Heading
MSM: men who have sex with men
PrEP: pre-exposure prophylaxis
PRISMA-ScR: Preferred Reporting Items for Systematic Reviews and Meta-Analyses extension for Scoping Reviews
RCT: randomized controlled trial
