# Supplementary material for: The Impact of Generative Conversational Artificial Intelligence on the Lesbian, Gay, Bisexual, Transgender, and Queer Community: Scoping Review
Source: J Med Internet Res. 2023 Dec 6;25:e52091. doi: 10.2196/52091 (PMC10733821; doi:10.2196/52091)
Supplement: Multimedia Appendix 2 [file jmir_v25i1e52091_app2.docx]

**Appendix 2. Detailed search strategy for each database utilized in the present scoping review.**

***PubMed/MEDLINE search strategy.***

("chatbot*"[All Fields] OR "virtual assistant*"[All Fields] OR "virtual agent*"[All Fields] OR "artificial intelligence"[All Fields] OR "generative AI"[All Fields] OR "conversational AI"[All Fields] OR "large language model*"[All Fields] OR "LLM"[All Fields] OR "LLMs"[All Fields] OR "ChatGPT"[All Fields] OR "Bard"[All Fields]) AND ("lgbt*"[All Fields] OR "homosexual*"[All Fields] OR ("queered"[All Fields] OR "queering"[All Fields] OR "queerness"[All Fields] OR "sexual and gender minorities"[MeSH Terms] OR ("sexual"[All Fields] AND "gender"[All Fields] AND "minorities"[All Fields]) OR "sexual and gender minorities"[All Fields] OR "queer"[All Fields] OR "queers"[All Fields]) OR "lesbian*"[All Fields] OR "bisexual*"[All Fields] OR "pansexual*"[All Fields] OR "intersex*"[All Fields] OR ("transgender persons"[MeSH Terms] OR ("transgender"[All Fields] AND "persons"[All Fields]) OR "transgender persons"[All Fields] OR "transgender"[All Fields] OR "transgendered"[All Fields] OR "transgenders"[All Fields]) OR "transsexual*"[All Fields] OR "men who have sex with men"[All Fields] OR "men having sex with men"[All Fields] OR "MSM community"[All Fields] OR "genderfluid"[All Fields] OR "genderqueer"[All Fields] OR "gender-diverse"[All Fields] OR "gender non-conforming"[All Fields] OR "nonbinary"[All Fields] OR "sexual and gender minorities"[All Fields] OR "sexual minority"[All Fields] OR "gender minority"[All Fields] OR "sexual orientation"[All Fields] OR "sexual identity"[All Fields] OR "gender identity"[All Fields] OR "gender expression"[All Fields] OR ("sexual behavior"[MeSH Terms] OR ("sexual"[All Fields] AND "behavior"[All Fields]) OR "sexual behavior"[All Fields] OR "sexual"[All Fields] OR "sexually"[All Fields] OR "sexualities"[All Fields] OR "sexuality"[MeSH Terms] OR "sexuality"[All Fields] OR "sexualization"[All Fields] OR "sexualize"[All Fields] OR "sexualized"[All Fields] OR "sexualizing"[All Fields] OR "sexuals"[All Fields]))

***Scopus search strategy.***

TITLE-ABS-KEY ((chatbot* OR "virtual assistant*" OR "virtual agent*" OR "artificial intelligence" OR "generative AI" OR "conversational AI" OR "large language model*" OR llm OR llms OR chatgpt OR bard) AND (lgbt* OR homosexual* OR queer OR lesbian* OR bisexual* OR pansexual* OR intersex* OR transgender OR transsexual* OR "men who have sex with men" OR "men having sex with men" OR "MSM community" OR genderfluid OR genderqueer OR gender-diverse OR gender-expanded OR "gender non-conforming" OR nonbinary OR "sexual and gender minorities" OR "sexual minority" OR "gender minority" OR "sexual orientation" OR "sexual identity" OR "gender identity" OR "gender expression" OR sexuality))

***Web of Science search strategy.***

ALL=((chatbot* OR “virtual assistant*” OR “virtual agent*” OR "artificial intelligence" OR “generative AI” OR “conversational AI” OR “large language model*” OR LLM OR LLMs OR ChatGPT OR Bard) AND (LGBT* OR homosexual* OR queer OR lesbian* OR bisexual* OR pansexual* OR intersex* OR transgender OR transsexual* OR “men who have sex with men” OR “men having sex with men” OR “MSM community” OR genderfluid OR genderqueer OR gender-diverse OR gender-expanded OR “gender non-conforming” OR nonbinary OR “sexual and gender minorities” OR “sexual minority” OR “gender minority” OR “sexual orientation” OR “sexual identity” OR “gender identity” OR “gender expression” OR sexuality))

***Embase search strategy.***

(chatbot* OR 'virtual assistant*' OR 'virtual agent*' OR 'artificial intelligence'/exp OR 'artificial intelligence' OR 'generative ai' OR 'conversational ai' OR 'large language model*' OR llm OR llms OR 'chatgpt'/exp OR chatgpt OR 'bard'/exp OR bard) AND (lgbt* OR homosexual* OR 'queer'/exp OR queer OR lesbian* OR bisexual* OR pansexual* OR intersex* OR 'transgender'/exp OR transgender OR transsexual* OR 'men who have sex with men'/exp OR 'men who have sex with men' OR 'men having sex with men'/exp OR 'men having sex with men' OR 'msm community' OR 'genderfluid'/exp OR genderfluid OR 'genderqueer'/exp OR genderqueer OR 'gender diverse' OR 'gender expanded' OR 'gender non-conforming'/exp OR 'gender non-conforming' OR nonbinary OR 'sexual and gender minorities'/exp OR 'sexual and gender minorities' OR 'sexual minority'/exp OR 'sexual minority' OR 'gender minority'/exp OR 'gender minority' OR 'sexual orientation'/exp OR 'sexual orientation' OR 'sexual identity'/exp OR 'sexual identity' OR 'gender identity'/exp OR 'gender identity' OR 'gender expression'/exp OR 'gender expression' OR 'sexuality'/exp OR sexuality)

***Google and Google Scholar search strategy***

(chatbot* OR “virtual assistant*” OR “virtual agent*” OR "artificial intelligence" OR “generative AI” OR “conversational AI” OR “large language model*” OR LLM OR LLMs OR ChatGPT OR Bard) AND (LGBT* OR homosexual* OR queer OR lesbian* OR bisexual* OR pansexual* OR intersex* OR transgender OR transsexual* OR “men who have sex with men” OR “men having sex with men” OR “MSM community” OR genderfluid OR genderqueer OR gender-diverse OR gender-expanded OR “gender non-conforming” OR nonbinary OR “sexual and gender minorities” OR “sexual minority” OR “gender minority” OR “sexual orientation” OR “sexual identity” OR “gender identity” OR “gender expression” OR sexuality)
